# Effects of evolutionary history on genome wide and phenotypic convergence in *Drosophila* populations

**DOI:** 10.1186/s12864-018-5118-7

**Published:** 2018-10-11

**Authors:** Mark A Phillips, Grant A Rutledge, James N Kezos, Zachary S Greenspan, Andrew Talbott, Sara Matty, Hamid Arain, Laurence D Mueller, Michael R Rose, Parvin Shahrestani

**Affiliations:** 1 Department of Ecology and Evolutionary Biology, University of California Irvine, Irvine, USA; 2 Department of Development, Aging, and Regeneration, Sanford Burnham Prebys Medical Discovery Institute, San Diego, USA; 3 Department of Biological Science, California State University Fullerton, 800 N State College Blvd, Fullerton, CA 92831 USA

**Keywords:** Experimental evolution, Evolutionary genomics, Evolutionary history, Adaptation

## Abstract

**Background:**

Studies combining experimental evolution and next-generation sequencing have found that adaptation in sexually reproducing populations is primarily fueled by standing genetic variation. Consequently, the response to selection is rapid and highly repeatable across replicate populations. Some studies suggest that the response to selection is highly repeatable at both the phenotypic and genomic levels, and that evolutionary history has little impact. Other studies suggest that even when the response to selection is repeatable phenotypically, evolutionary history can have significant impacts at the genomic level. Here we test two hypotheses that may explain this discrepancy. *Hypothesis* 1: Past intense selection reduces evolutionary repeatability at the genomic and phenotypic levels when conditions change. *Hypothesis* 2: Previous intense selection does not reduce evolutionary repeatability, but other evolutionary mechanisms may. We test these hypotheses using *D. melanogaster* populations that were subjected to 260 generations of intense selection for desiccation resistance and have since been under relaxed selection for the past 230 generations.

**Results:**

We find that, with the exception of longevity and to a lesser extent fecundity, 230 generations of relaxed selection has erased the extreme phenotypic differentiation previously found. We also find no signs of genetic fixation, and only limited evidence of genetic differentiation between previously desiccation resistance selected populations and their controls.

**Conclusion:**

Our findings suggest that evolution in our system is highly repeatable even when populations have been previously subjected to bouts of extreme selection. We therefore conclude that evolutionary repeatability can overcome past bouts of extreme selection in *Drosophila* experimental evolution, provided experiments are sufficiently long and populations are not inbred.

**Electronic supplementary material:**

The online version of this article (10.1186/s12864-018-5118-7) contains supplementary material, which is available to authorized users.

## Background

The combination of experimental evolution and next generation sequencing has become established as a powerful means for studying the genetics of adaptation and testing major tenets of population genetic theory [1, 2]. Studies featuring populations of fruit flies, *Drosophila melanogaster*, suggest that adaptation in sexually reproducing populations is fueled by selection on standing genetic variation, and is largely characterized by a lack of genetic fixation [3–9]. The apparent lack of fixation is even seen in long-term experiments nearing a thousand generations of selection [10]. Moreover, work with outcrossing populations of *Saccharomyces cerevisiae* has shown that adaptation is still primarily driven by standing genetic variation even at much larger effective population sizes than what is currently seen in experiments featuring *D. melanogaster* [11]. At present, the underlying genetic architecture of adaptation in these experiments is an area of active study and debate, but these broad results regarding sexually reproducing populations are largely consistent across a variety of independent experiments [1, 2].

In accordance with these findings, evolution in outbred populations is rapid, and highly repeatable when newly derived *D. melanogaster* populations are subjected to the same selection regimes as long-standing populations [12, 13]. It takes only dozens of generations for newly derived populations to converge on long-standing populations at both the genomic and phenotypic levels, even when long-standing populations have previously undergone hundreds of generations of selection [12, 13]. These findings suggest that phenotypes and patterns of genetic variation are primarily shaped by most recent selection regime, and that evolutionary history, prior to the recent selection regime, has little discernible impact. However, this runs contrary to evidence from experimental evolution work using *Drosophila subobscura* derived from wild populations at contrasting European latitudes [14]. Those findings indicate that evolution is predictable at the phenotypic level, but differences in where source populations originate can have significant effects on outcomes at the genetic level, suggesting that evolutionary history does play a role when it comes to repeatability at the genomic level. The idea that the degree to which populations return to ancestral phenotypic values and allele frequencies is at least in part contingent on evolutionary history is also supported by reverse experimental evolution studies [3, 15]. However, it should be noted that the authors of these reverse experimental evolution studies were unable to rule out the possibility that complete reversion would have occurred in all populations if given more time.

A possible resolution to why evolutionary history appears to play a role in some experiments but not others follows from Graves et al. [13]. In addition to the aforementioned results, the authors found evidence that more intense selection regimes lead to significantly greater losses of genetic variation compared to milder selection regimes. While they did not observe strong evidence of fixation within any of the populations studied, fixation seemed at least possible provided a sufficiently intense selection regime. Therefore, the finding that phenotypes and patterns of genetic variation are almost exclusively shaped by most recent selection regime in Burke et al. [12] and Graves et al. [13] could be due to the fact that while selection for accelerated for development is intense, it is perhaps not sufficiently intense to bring about the sort of changes necessary to impact future evolutionary trajectories. Presumably, stronger selective pressures could potentially result in widespread fixation of alleles favored by such selection. In these cases, given that adaption in sexual experimental evolution is primarily fueled by standing genetic variation, the widespread fixation could have significant impact on how experimental populations respond to new selective pressures, and their ability to revert to ancestral states when moved back to ancestral conditions (i.e. the evolutionary repeatability at the genomic level typically seen in this sort of work would be reduced). The failure to find fixation in the populations selected for accelerated development in Burke et al. [12] and Graves et al. [13] also does not necessarily mean the genomic changes brought about by that selection regime will not negatively impact future evolutionary repeatability. For instance, if adaptation in this sort of system involves shifts in equilibrium frequencies at many sites across the genome as a result of antagonistic pleiotropy [16, 17], intense selection may drive frequencies to attractor states that constrain adaptation when conditions change.

We do not have data from experiments where populations exposed to selection for accelerated development were moved to new selection regimes. Instead, we explore adaptation to new selection regimes with a set of populations we have that were previously subjected to very intense selection for desiccation resistance. Using these populations, we test the following hypotheses: Hypothesis (1) past intense selection reduces evolutionary repeatability at the genomic and phenotypic levels when conditions change. Hypothesis (2) previous intense selection does not reduce evolutionary repeatability at the genomic and phenotypic levels, in the absence of other factors such as inbreeding or chromosomal rearrangement.

As mentioned above, we test these hypotheses using a group of *D. melanogaster* populations that were historically subjected to intense selection for desiccation resistance. In the past, we have shown that populations subject to selection for desiccation resistance are useful material for analyzing the mechanistic foundations of adaptation in lab fruit flies as they produce highest levels of phenotypic differentiation between selected populations and controls observed within our experimental system [18–24]; keeping in mind that our system includes the populations selected for accelerated development featured in Burke et al. [12] and Graves et al. [13]. The extreme phenotypic shifts brought about by this particular selection regime are attributed to the fact the regime involves using environments so inimical to survival only a small percentage (10–20%) of each generation survives [18–21]. We have called this intense selection paradigm “culling selection” in the past, and it represents one of the most extreme protocols used in *Drosophila* experimental evolution [25]. Lastly, unlike the populations selected for accelerated development previously discussed, we have extensive data on what these populations look like after selection for desiccation resistance was no longer being imposed.

Specifically, to test our hypotheses we examine patterns of phenotypic and genomic differentiation in two five-fold replicated stocks, TSO _1–5_, and TDO _1–5_, known as C and D respectively during active selection, which were first described in Rose et al. [18]. The D populations were intensely selected for desiccation resistance for about 260 generations, and afterward were renamed as TDO, and maintained on a 21 day (T for “Three-week”) relaxed culture selection for the past ~ 230 generations. The C populations were moderately selected for starvation resistance for about 260 generations in parallel with the D populations, serving as controls for the D populations, and were later renamed as TSO, and maintained under the same culture selection regime as the TDO populations. The TSO and TDO populations were all placed under relaxed culture selection during the same generations. The extreme functional differentiation (i.e. carbohydrate content, water loss rates, and water content) previously seen between these two groups was achieved using environments so inimical to survival that only a small percentage (10–20%) of each generation survives selection [18–21] With Hypothesis 1, large impacts of evolutionary history are in fact due to exposure to intense selection. If this hypothesis is correct, we would expect to find lingering phenotypic and significant genomic differentiation between the TDO and TSO populations even after ~ 230 generations of relaxed selection in the former. If Hypothesis 2 is correct, we should not find such differentiation.

## Results

### Phenotypic results

#### Mortality and mean longevity

**Fig. 1:**
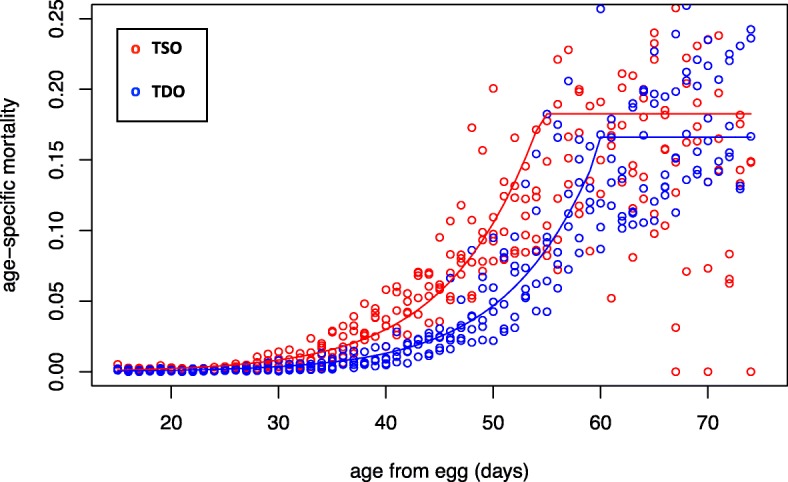
Age-specific mortality rates across the five TDO and TSO replicates shown in blue and red open circles respectively. The data was fitted by a two-stage, three-parameter Gompertz model. Fitted lines for the TDO and TSO populations are shown in blue and red respectively. TDO’s are living significantly longer than the TSO’s

Mortality rates were measured in the TSO and TDO populations (Additional file 1). From the Gompertz model fit to our mortality data, A is the age-independent parameter which gives a measure of the baseline mortality rate. α is the age-dependent parameter which gives a measure of the rate of aging. The TDO populations have lower values for the parameters A and α compared to the TSO populations. These differences are significant for A (*p* = 0.0001; Fig. 1; Additional file 2: Table S2 and see Figure S1 for survivorship plots) but are not significant for α (*p* = 0.945; Fig. 1; Additional file 2: Table S2). In addition, the TDO populations show a greater break-day (bd) compared to the TSO populations (*p* < 0.0001; Fig. 1; Additional file 2: Table S2).

**Fig. 2:**
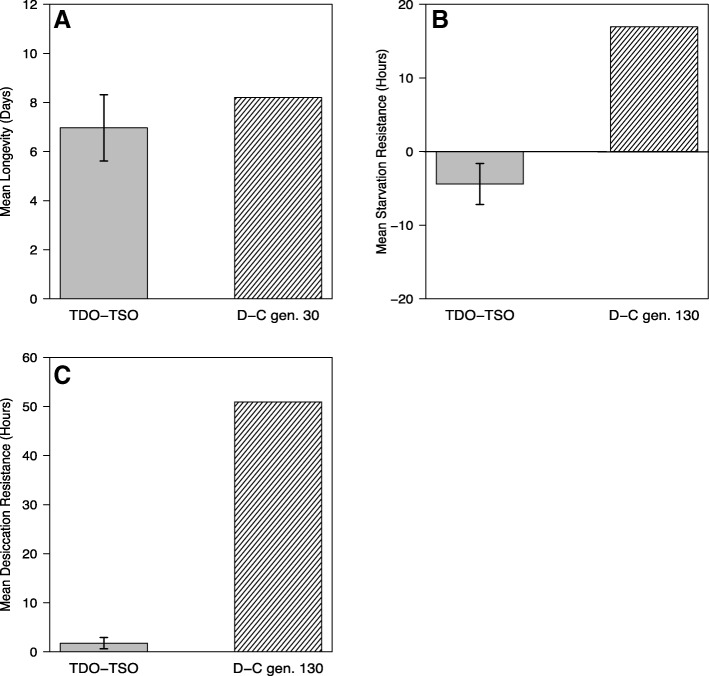
Historical and current starvation resistance, desiccation resistance and mean longevity data from females in the desiccation selected and control lines. **a** Difference in average longevity between the selected and control populations. Difference in mean longevity was highest early in desiccation selection (near generation 30) [18] and then decreases to near zero toward the end of selection [21]. **b** Difference in mean survival time in a starvation environment of flies at age 15 days from egg. The TSO and TDO populations are not significantly different in starvation resistance. However, starvation resistance was significantly different during generation 130 of active desiccation selection [20, 22]. **c** Difference in mean survival time in a desiccation environment of flies at age 15 days from egg. Desiccation resistance differences were highest toward the end of selection (generation 200) and have since returned to close to zero [20, 21]. Error bars are mean ± 1 SEM

When analyzing mean longevity, the TDO populations live ~ 7 days longer than the TSO populations (*p* = 0.0009; Additional file 2: Table S3). These significant differences are observed in both males and females. As seen in Fig. 2a, the observed difference in mean longevity is comparable to the peak difference in mean longevity observed when the populations were under directional selection (when they were maintained as C’s and D’s).

#### Development time: larvae to adult

**Fig. 3:**
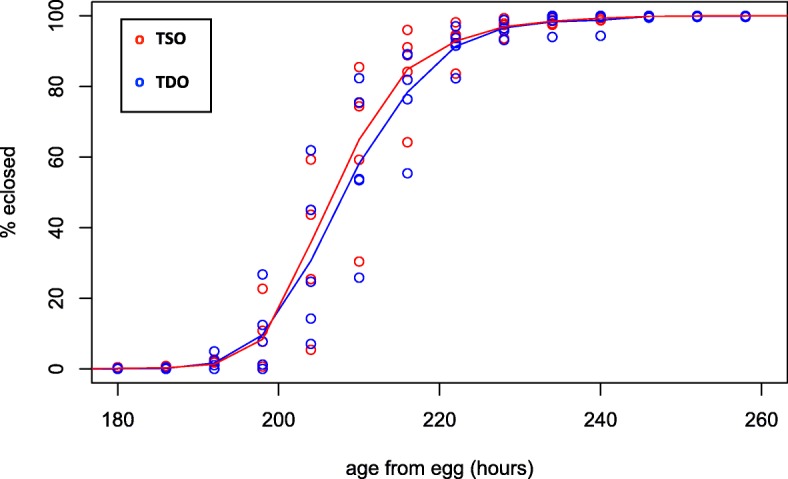
Time to eclosion in the TDO and TSO populations. Points represent the percentage of the total cohort of flies eclosed each collection interval for each replicate. Lines represent averages across replicates. TSO populations are represented by red lines and open red circles, while the TDO populations are represented by blue lines and open blue circles. The TDO’s are statistically converged upon the TSO’s for this development time measure (*p* = 0.66)

Larvae to adult development time was measured in the TSO and TDO populations (Additional file 3). The TDO populations take about 1 h longer to eclose from pupa compared to the TSO populations, however this difference is not significant (*p* = 0.66; Fig. 3; Additional file 2: Table S4).

#### Adult age-specific fecundity

**Fig. 4:**
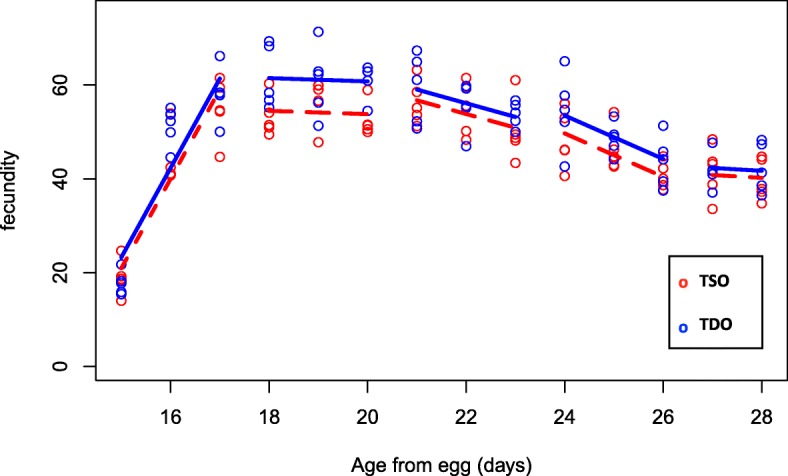
Adult age-specific fecundity from the TDO and TSO populations. Open red circles and dashed red lines represent average eggs laid per female per day as a function of age in the five TSO populations. Open blue circles and solid blue lines represent the five TDO populations. TDO populations have significantly higher fecundity in the second interval (*p* = 0.021). All other intervals are not significant (*p* > 0.05)

Age-specific fecundity was monitored in the TDO and TSO populations (Additional file 4). The TDO populations show a greater number of eggs laid per surviving female (m_x_) compared to the TSO populations in the second interval (days 18–20 from egg) (*p* = 0.021; Fig. 4; Additional file 2: Table S5). This is the interval just prior to these populations’ reproductive window (days from egg 20–21). The reproductive window is the period when eggs are used from these populations for the next generation. All other intervals from the analysis are not significant (*p* > 0.05).

#### Fungal-resistance

**Fig. 5:**
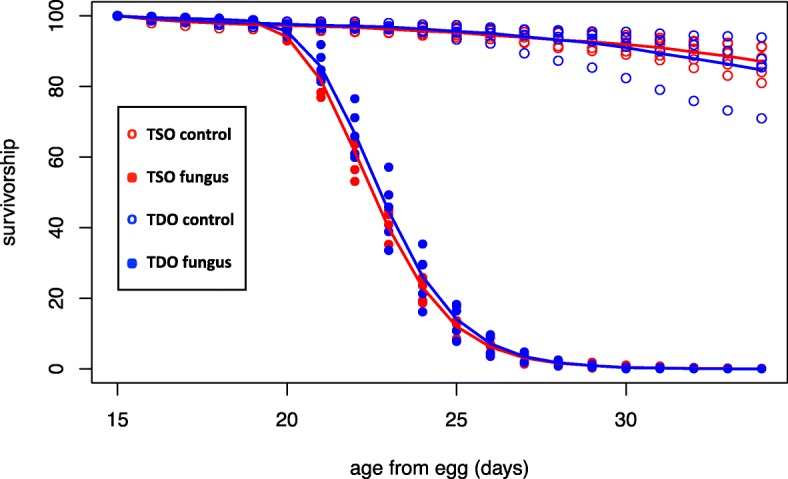
Adult survivorship (1_×_) from the TDO and TSO fungal resistance experiment. The control populations are represented by open points and the populations exposed to fungus are represented by closed points. TSO and TDO points are shown in red and blue respectively. Trend lines show averages across all replicates and across three experiments. Fungal resistance is not significantly different between the TDO and TSO populations. (*p* = 0.123)

Mortality after exposure to the fungus *Beauveria bassiana* was monitored in the TDO and TSO populations (Additional file 5). No difference was observed in the ability of these populations to survive after exposure to the fungus (*p* = 0.123; Fig. 5).

#### Starvation resistance

The average survival time under starvation conditions was monitored in the TDO and TSO populations (Additional file 6). The average survival time during starvation, or starvation resistance, for the five TSO populations is 73.54 h, and for the five TDO populations, it is 69.05 h (Fig. 2b). This 4.49 h difference in starvation resistance is not statistically significant (*p*-value = 0.152; Additional file 2: Table S6). In contrast, there was a notable difference in starvation when the D populations were being actively selected for desiccation resistance (Fig. 2b).

Some might argue that our failure to detect any differences in starvation resistance may be due to a lack of statistical power, but this seems unlikely. Historically the difference in starvation resistance for the D population minus the C populations was over 10 h. In this study the observed difference between the TSO and TDO populations was 4.49 h, which is not statistically significant. However, this test had the ability to detect a difference of 5.57 h. Thus, differences even close to the historical C and D difference should have been easily detected. If the true starvation resistance of the TDO population had been 83.4 h rather than 69, giving TDO’s a 10 h advantage over TSO’s, the probability of detecting that difference would have been 0.88. If the TDO populations had only 5 h starvation resistance advantage the chance of detecting that differences drops 0.31.

#### Desiccation resistance

The average survival time during desiccation was monitored in the TDO and TSO populations (Additional file 7). The desiccation resistance for the five TSO populations is 13.26 h, and for the five TDO populations, it is 15.04 h (Fig. 2c). This 1.78 h difference in survival time is not statistically significant (*p*-value = 0.164; Additional file 2: Table S7). As seen in Fig. 2c, this observed difference in the TDO and TSO populations is nearly 30 times smaller than what was seeing during the height of selection for desiccation resistance.

Once again, our failure to detect lingering desiccation could be attributed to a lack of statistical power. We once again argue that this is unlikely. Historically the difference in desiccation resistance for the D population minus the C populations was about 50 h. In this study the observed difference between the TSO and TDO populations was − 1.78 h, which is not statistically significant. However, this test had the ability to detect a difference of 2.67 h. If the true TDO desiccation time had been only 3 h greater than the TSO time the chance of detecting the difference would be 0.60. If the true TDO desiccation time had been 5 h greater the chance of detecting the difference increases to 0.96.

#### Cardiac arrest rates

The rates of cardiac arrest after electrical pacing were monitored in the TSO and the TDO populations (Additional file 8). The five TSO populations had an average cardiac arrest rate of 27.6%. Whereas, the five TDO populations had an average cardiac arrest rate of 25.78%. Similar to the starvation resistance and desiccation resistance, this small difference between the two sets of populations was not statistically significant (Additional file 2: Figure S2, *p*-value = 0.598).

### Genomic results

#### Heterozygosity and F_ST_

**Fig. 6:**
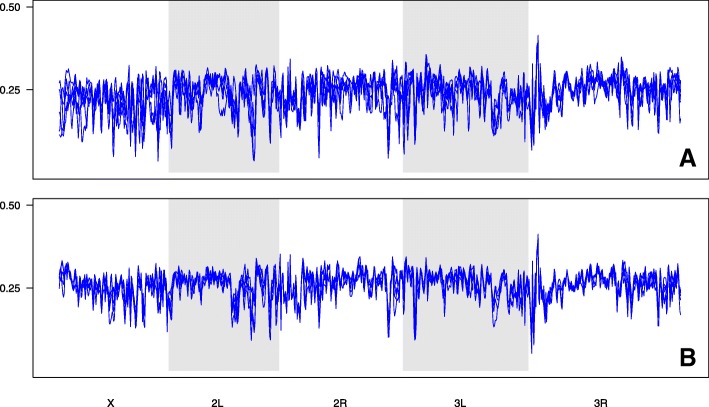
Heterozygosity in the TSO (**a**) and TDO (**b**) populations plotted over 150-kb windows across all major chromosome arms. All replicates are shown for each population

We do not see any large regions where heterozygosity has been completely expunged, and this result is robust to reductions in window size (Fig. 6, Additional file 2: Figures S2 and S3). However, there are some notable depressions consistent across replicates that may be indicative of soft sweeps. Mean heterozygosity in the TSO populations ranges from 0.24 to 0.26, and 0.26 to 0.27 in the TDO populations (Additional file 2: Table S8). Based on a t-test comparing the two sets of means, heterozygosity is significantly higher in the TDO populations (*p*-value = 0.001). Mean F_ST_ in the TSO populations is 0.04 and 0.07 in the TDO populations, which indicates there is a high degree of similarity between replicates.

#### SNP differentiation

**Fig. 7:**
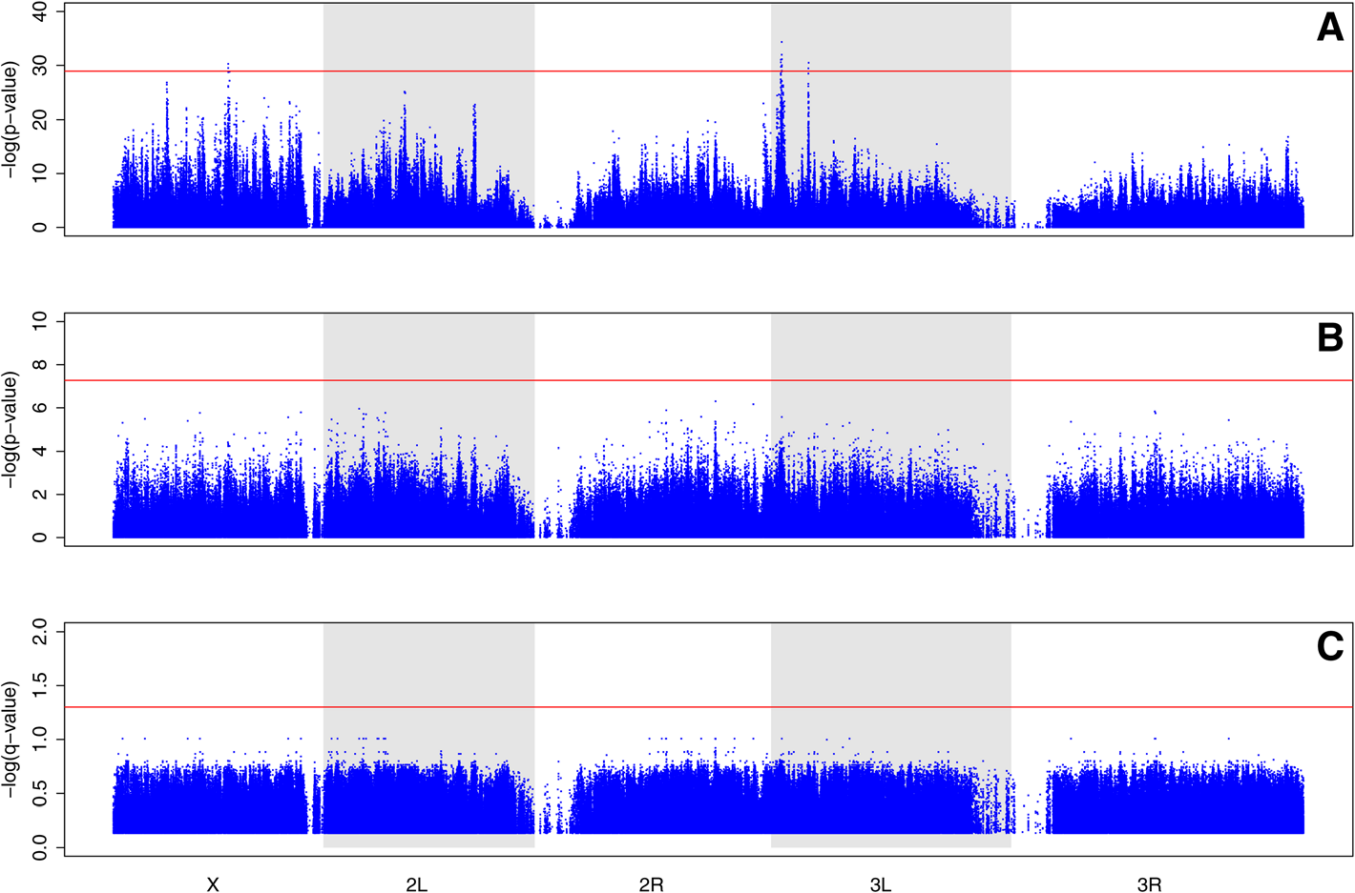
Results from statistical test comparing SNP frequencies in the TDO and TSO populations. **a** Results from CMH tests plotted along all major chromosome arms as –log(*p*-values). Our permutation derived significance threshold is shown in red. **b** Results from quasibinomial GLM approach plotted as −log(*p*-values), and our Bonferroni corrected significance threshold is shown in red. **c** Results from quasibinomial GLM converted to *q*-values, and plotted –log(*q*-values). A 0.05 false discovery rate threshold was used, as shown in red

We find little evidence of SNP (single nucleotide polymorphism) differentiation between the TDO and TSO populations (Fig. 7). Based on our Cochran-Mantel-Haenszel (CMH) test results, we find a total of 17 sites with *p*-values that exceed our permutation derived significance threshold (Fig. 7a). These 17 sites correspond to three regions, two on chromosome 3 L and one on the X chromosome. However, we find no signs of significant SNP differentiation using the quasibinomial GLM method. This is true using both the Bonferroni correction, and the less conservative *q*-value approach to correct for multiple comparisons (Fig. 7b–c).

Within the significantly differentiated regions detected using the CMH test, we find a total of seven genes (Table 1). Six of the seven genes are located on chromosome arm 3 L, while the remaining gene is located on chromosome X. For genes *CR42860*, *CR45802*, and *CR34047*, there is little to no information about their molecular and biological functions. Gene CG42355 is associated with sperm chromatin condensation, but not much else is presently known. Genes *sallimus* (*sls*) and *zormin* have been well documented to be associated with the development of the striated muscle sarcomeres [26–28]. *Sls* expression is necessary for myoblast fusion, and the inevitable development of myoblasts into muscle fibres. Sallimus, protein derived from the *sls* gene, aides in aligning thin filaments side-by-side and in anti-parallel direction, which nucleates Z-disc formation in developing myofibrils [26]. Sallimus also binds to thin filaments (i.e. actin), aiding in balancing the two halves of the sarcomere. Protein zormin can be also be found near the Z-disc and M-line of the muscles [28]. These filaments connect the Z-disc with the ends of the thick filaments. The size of these proteins, and the extensibility of their binding, affects the elasticity and stiffness of muscles [27]. These properties dictate muscle contraction, stiffness, and performance. The final gene, *CG32649*, located on the X chromosome is associated with ubiquinone biosynthesis and mitochondrial electron transport. There are two human orthologs, *COQ8A* and *COQ8B*, linked to *CG32649*. Mutations at these two ADCK genes can lead to primary coenzyme Q-10 deficiency and nephrotic syndrome, respectively [29, 30].

**Table 1:** Genes located in regions found to be significantly differentiated based on our CMH test comparing SNP frequencies in the TDO and TSO populations

Gene	Location	Association	Molecular Function	Biological Process
CG42355	3L: 2037371-2038224.	Unknown	Unknown	Sperm chromatin condensation.
sls (Sallimus)	3L: 2039681-2115611	Protein necessary for myoblast fusion; determinant of resting elasticity of striated muscle sarcomeres (myofibril stiffness); regulates mitochondrial respiration in sarcomere	Structural constituent of muscle; Actin binding; Protein binding.	Chromosome organization; skeletal muscle organ development; regulation of immune system process; mesoderm development; chromosome condensation; locomotion; somatic muscle development; myotube differentiation; visceral muscle development; striated muscle tissue development; regulation of multicellular organismal process.
CR42860	3L: 2088166-2089626	Unknown	Unknown	Unknown
Zormin	3L: 2117466-2151700	Found in the Z-disc and the M-line of muscles. Affects elasticity and stiffness of sarcomeres.	Protein binding; actin binding	
CR45802	3L: 2118498-2119567	Unknown	Unknown	Unknown
CR34047	3L: 5098376-5099795	Unknown	Unknown	Unknown
CG32649	X: 12898768-12901114	Ubiquinone biosynthesis; CoQ8A and CoQ8B human orthologs	Protein kinase activity.	Mitochondrial electron transport, ubiquinol to cytochrome c.

#### Migration simulations

Given how long these populations have been maintained in the lab, some might argue that the lack of SNP differentiation between the TDO and TSO populations is perhaps due to low frequency accidental migration events. To test this idea, simulations were performed using SNP frequencies in the ACO and CO populations featured in Burke et al. [12] and Graves et al. [13] as a starting point (See Methods for details). Data from these populations were used as a proxy for what differentiation between the TDO and TSO populations might have looked like during the height of selection for desiccation resistance. We simulated 230 generations of neutral evolution with varying levels of migration (two, six and ten events per generation), and looked at how our ability to detect differentiated sites using the quasibinomial GLM approach was impacted (Additional file 2: Table S9). We looked at the number of significantly differentiated sites using both a Bonferroni correction (our most stringent method) and the *q*-value approach to correction for multiple comparisons (or most liberal method).

Applying this test to observed SNP frequencies in the ACO and CO populations, we detect 162 sites when we use the Bonferroni method for correction for multiple comparisons, and ~ 425 k when we use the less stringent *q*-value approach. We find that in our simulated data sets, migration does reduce the number of significantly differentiated sites detected. For instance, the number of sites that are significant after a Bonferroni correction is less than three for each of the scenarios we looked at (Additional file 2: Figure S5). However, this is still greater than the zero sites detected between the TDO and TSO populations. Using the *q*-value method, we find that migration rates of two and six similarly reduce the number of detected sites by ~ 270–300 k, and a migration rate of 10 reduces the number of detected sites by ~ 400 k. However, even in our most extreme scenario with 10 migration events every generation, we still detect ~ 20 k differentiated sites compared to zero in the TSO and TDO populations (Additional file 2: Figure S6). Given that it is incredibly unlikely there was anything approaching the equivalent of 10 migrations events per generation between the TDO and TSO treatments, these findings suggest that the lack of differentiation between the TDO and TSO populations cannot be easily attributed to accidental migration events.

## Discussion

Our results indicate that ~ 230 generations of relaxed selection were enough for the previously desiccation selected TDO populations to largely converge on the TSO controls phenotypically and perhaps genomically. Aside from longevity and one interval for fecundity, we find no signs of phenotypic differentiation between the TDO and TSO populations for any of the characters measured. Most notably, the TDO populations do not show any signs of significantly enhanced survival in desiccating environments compared to the TSO populations, despite extreme differences in desiccation resistance prior to the relaxation of selection [18–20, 23]. There is also no longer any evidence of increased starvation resistance in the TDO populations, which was a trait previously found to be correlated with enhanced desiccation resistance [20]. Next, we found no differences in fungal resistance and development time, which are traits we would also have expected to be impacted by selection for desiccation resistance. Lastly, significant differences in female fecundity were also limited to a single window spanning day 18 to day 20 from egg (day 9–11 from eclosion). Chippindale et al. [31] found that D populations had significantly higher fecundity compared to the C populations. However, their experiment only measured early fecundity (day 3–5 from eclosion). It is also worth noting that the observed phenotypic reversion under control conditions may not be due entirely to shifts in allele frequencies. Instead, they may be the product of gene by environment interactions as has been well characterized in the quantitative trait locus literature [32–34].

Longevity is the only trait we studied that still shows clear signs of phenotypic differentiation between the TDO and TSO populations. This arises notwithstanding the absence of genomic differentiation detected between these two sets of populations. There are two ways to resolve this paradox at the level of statistical analysis. First, it is conceivable that the longevity differentiation arose by chance alone, given that we compared these two sets of populations for multiple phenotypes. However, if we perform a Bonferroni correction on the threshold for statistical significance making it 0.003, the observed *p*-value of 0.001 remains significant for longevity. Second, if we grant the point that there is a significant longevity difference, then the failure to detect extensive genomic differentiation becomes an issue. However, if we consider the genomic analysis of differentiation between A and C populations of Graves et al. [13], we find that there is a general reduction in the ability of genomic analysis to detect differentiation when only ten populations total are compared as two groups of five. In Graves et al. [13], thousands of differentiated sites were detected when comparing all ten A-type populations to all ten C-type populations, compared to hundreds of sites when groups of five were compared to one another. This finding is also supported by theoretical studies examining the power of evolve and re-sequence studies to detect causal variants [35, 36]. As we are limited to five replicates per treatment in this study, it is possible that we simply do not have the statistical power to detect the genomic differentiation underlying this residual phenotypic differentiation.

Previous work has shown that selection for increased longevity is associated with increased desiccation resistance [37, 38], and furthermore selection for increased desiccation resistance was associated with increased longevity [18]. Further work with sustained selection for desiccation resistance revealed a more complex relationship, with the greatest benefits for longevity accruing at intermediate levels of increased desiccation resistance [21, 22]. It is surprising and perhaps noteworthy that the longevity difference between the TDO and TSO populations is similar to the longevity difference that they exhibited at their peak of differentiation for this character, particularly for females [18, 31]. Furthermore, when desiccation selection proceeded to very high levels of desiccation resistance in the D (ancestral to TDO) populations, their differentiation for longevity relative to the C (ancestral to TSO) populations actually fell from this peak. In the case of the present TDO and TSO populations, the differentiation of desiccation is now gone, at least at the level of statistical detectability. Yet the longevity difference has returned to its former peak level. One possible explanation for this is that the T culture regime may favor increased longevity, or moderately increased longevity is at least not selected against by any type of antagonistic pleiotropy. However, we have no way of distinguishing between these hypotheses at the present time.

Although our selection protocol for desiccation resistance is relatively extreme, compared to other selection regimes we have used [39] we do not find any clear evidence of it having a lasting impact on levels of genetic variation. Specifically, our analysis did not yield any evidence that being subjected to intense selection in the past has led to widespread fixation in the TDO populations. As such, our findings suggest that even when a moderately outbred experimental population’s evolutionary history involves prolonged periods of intense selection, it does not have irreversible effects on levels and patterns of genetic variation. This lack of fixation also indicates that the rapid and highly repeatable evolution from standing genetic variation seen in Graves et al. [13] should still be possible when populations previously subjected to periods of intense selection are exposed to new conditions.

Unfortunately, we cannot directly compare current levels of SNP differentiation between the TDO and TSO populations to what they were during the height of TDO selection for desiccation resistance. However, given the levels of phenotypic differentiation between the two groups during the height of selection [18–22], we can reasonably suggest that total SNP differentiation between the two groups during this period was likely comparable to the dozens to hundreds of differentiated sites typically detected in *Drosophila* experimental evolution studies [4–9]. We also believe the findings of Burke et al. [12] and Graves et al. [13] in particular support this rationale as they were performed with population from the same system. Burke et al. [12] shows that selection for accelerated development generates levels of phenotypic differentiation between selected populations and controls approaching what is seen during the height of selection for desiccation resistance, and Graves et al. [13] shows that this is accompanied by wide-spread SNP differentiation when compared to controls and an overall reduction in levels of genetic variation. As such, the present lack of SNP differentiation between the TDO and TSO populations can be interpreted as evidence that ~ 230 generations of relaxed selection were enough to reduce obvious signs of genomic differentiation between the two groups.

CMH tests comparing SNP frequencies between the two groups of populations did yield some significantly differentiated sites. However, this was limited to 17 sites compared to the dozens to potentially thousands of differentiated sites likely present during the height of selection, as seen in other experimental evolution studies [4–9]. There were a total of seven genes associated with these sites, but none of these candidate genes had clear connections to desiccation resistance (See Results for details). Additionally, the quasibinomial GLM approach to detecting significantly differentiated SNP’s advocated by Wiberg et al. [35] did not detect any significant SNP differentiation between the TDO and TSO populations. Given the issues with the CMH test documented in by Wiberg et al. [40], the discrepancy between the two tests casts some degree of doubt on whether or not the few sites detected using the CMH test are truly differentiated. As such, we conclude that ~ 230 generations of relaxed selection were enough to largely eliminate the signs of meaningful SNP differentiation likely present between the TDO populations and their control during the height of selection. This further suggest that a past history of intense selection does not necessarily reduce evolutionary repeatability at the genomic level in *Drosophila* experimental evolution.

Our genomic findings about the role of evolutionary history in shaping patterns of genetic variation are not entirely conclusive however. For instance, while we have phenotypic data for the TDO populations prior to the relaxation of selection, we do not have any genomic data from this period because original work with these population pre-dated affordable genome wide sequencing. As such, we cannot directly show that relaxing selection resulted in significant shifts in patterns of genetic variation. We also cannot directly compare current levels of SNP differentiation between the TDO and TSO populations to what they were during the height of selection for desiccation resistance as previously mentioned. Lastly, we acknowledge that as is often the case with studies combining experimental evolution and pooled sequencing, our results were perhaps impacted by some pronounced underlying haplotype structure. Exploring this possibility is undoubtedly a worthwhile venture but falls beyond the scope of our study at present. However, assuming past experimental evolution studies featuring genome-wide comparisons between experimentally evolved *Drosophila* populations are broadly applicable, these results nevertheless suggest patterns and levels of genetic variation and differentiation in *Drosophila* experimental evolution are not impacted by sustained strong selection so as to eliminate the potential for evolutionary repeatability in response to subsequent selection.

## Conclusion

Cumulatively, our findings suggest that past bouts of extreme selection do not negate the potential for evolutionary repeatability at the genomic and phenotypic levels in response to future selection in *Drosophila* experimental evolution. While we are able to detect some signs of genetic differentiation and residual differences in mean longevity when comparing the TDO and TSO populations, it is nothing on the order of what is usually found between selected and control populations in *Drosophila* experimental evolution [4–9]. And there is no reason to believe these differences would dramatically impact how these populations respond to future selection. As such, we conclude that past intense selection does not necessarily eliminate the possibility of evolutionary repeatability in response to future selection in experimental evolution studies featuring sexually reproducing populations, provided the duration of these experiments is sufficiently long and populations are not inbred at any point.

## Methods

### Populations

This experiment used large, outbred lab populations (effective populations size of ~ 1000 [41]) of *Drosophila melanogaster* derived from a population sampled by P.T. Ives from South Amherst, Massachusetts (Ives, 1970). The experimental stocks used in this study were derived from a set of five populations that had been selected for late reproduction (O_1–5_). The O_1–5_ populations were derived from the Ives stock in February 1980 [42]. In 1988, two sets of populations were derived from the O_1–5_ populations. One set (D_1–5_) were selected for desiccation resistance while the other set (C_1–5_) were maintained to control for desiccation resistance selection. The C_1–5_ populations were handled like the D_1–5_ populations, except flies were given nonnutritive agar instead of desiccant [18]. In 2005, these populations were relaxed from selection and kept on a 21-day culture regime to the present day. Under this new regime, the D populations have been renamed to TDO, and the C populations to TSO. In total, the TDO populations underwent ~ 260 generations of selection for desiccation resistance, and ~ 230 generations of relaxed selection.

Populations were reared on a banana-molasses diet for stock maintenance and for experimental assays. The banana-molasses media is composed of the following ingredients per 1 L distilled H_2_0: 13 g Apex® Drosophila agar type II, 120 g peeled, ripe banana, 40 mL light Karo® corn syrup, 40 mL dark Karo® corn syrup, 50 mL Eden®organic barley malt syrup, 32 g Red Star® active dry yeast, 2 g Sigma-Aldrich® Methyl 4-hydroxybenzoate (anti-fungal), and 42 mL EtOH. Stocks are maintained on a 24-h light cycle and kept at room temperature (24 °C ± 1 °C).

### Phenotypic assays

#### Mortality and mean longevity

For this assay, the TDO and TSO populations were reared in eight dram polystyrene vials with ~ 6 mL of food, an egg density of 60–80 eggs and given 14 days to develop. Adult flies from each replicate were transferred on day 14 from egg to three, six-liter acrylic plastic cages with ~ 1000 flies per cage (~ 3000 flies per replicate). Flies were given fresh food daily, and every 2 weeks flies were transferred to clean cages using light CO_2_ anesthesia. Individual mortality was assessed every 24 h, the flies were sexed at death, and the exact cohort size was calculated from the complete recorded deaths. Total cohort size across all replicates from both regimes was ~ 30,000 flies.

Mean longevity was analyzed using a linear mixed-effects model (LME) in the R-project for statistical computing (www.R.project.org) [43]. The model used for the data is described as follows: Let *y*_*ijkm*_ be the longevity for regime – *i* (*i* = 1 (TDO) or 2 (TSO)), sex-*j* (*j* = 1 (female), 2(male)), population – *k* (*k* = 1,.., 10) and individual – *m* (*k* = 1,.., n_*jk*_). A LME model for longevity is,

$$ {y}_{ijkm}=\alpha +{\delta}_i{\beta}_i+{\delta}_j\gamma +{\delta}_i{\delta}_j\pi +{b}_k+{\varepsilon}_{ijkm} $$

where *δ*_*s*_ = 0, if *s* = 1, and 1 otherwise, and *b*_*k*_ and ε_*ijkm*_ are assumed to be independent random variables with a normal distribution with zero mean and variances ${\sigma}_1^2$ and ${\sigma}_2^2$ respectively.

Mortality rates from the TDO and TSO populations were analyzed using a two-stage, three-parameter Gompertz model. The Gompertz model and its variants describe the change in instantaneous mortality rates with age. The chance of dying between day *t* and *t* + 1, *q*_*t*_, was estimated as,

$$ {q}_t=1-\frac{p_{t+1}}{p_t}\ \mathrm{where} $$

$$ {p}_t=\left\{\begin{array}{c}\exp \left\{\frac{A\left[1-\exp \left(\alpha t\right)\right]}{\alpha}\right\}\  if\ t\le bd\\ {}\mathit{\exp}\left\{\frac{A\left[1-\exp \left(\alpha bd\right)\right]}{\alpha }+ Aexp\left(\alpha bd\right)\left( bd-t\right)\right\} if\ t> bd\end{array}\right. $$

where *bd* is the break day or the age at which mortality rates transition from a Gompertz dynamic to a plateau.

With this model we let *y*_*ijkt*_ be the mortality from selection regime-*i* (*i* = 1 (TDO), 2 (TSO)), sex-*j* (*j* = 1 (female), 2(male)) and population-*k* (*k* = 1, 2, …, 10), at age-*t*. Random variation arises due to both population effects and individual variation. Consequently, the mortality of adults from selection regime-*i*, sex-*j*, and population-*k*, at time-*t* is *y*_*ijkt*_ = *f*(**φ**_*ijk*_,*t*) + ε_*ijkt*_, where **φ**_*ijk*_ is the vector of parameters, (*A*_*ijk*_, α_*ijk*_, *bd*_*ijk*_), and,

$$ {A}_{ijk}={\pi}_1+{\delta}_i{\beta}_{1i}+{\delta}_j{\gamma}_1+{b}_{1k} $$

$$ {\alpha}_{ijk}={\pi}_2+{\delta}_i{\beta}_{2i}+{\delta}_j{\gamma}_2+{b}_{2k} $$

$$ {bd}_{ijk}={\pi}_3+{\delta}_i{\beta}_{3i}+{\delta}_j{\gamma}_3+{b}_{3k} $$

where δ_*s*_ = 0, if *s* = 1 and 1 otherwise. The within population variation, ε, is assumed to be normally distributed with a zero mean. This variation increases with age so we assumed that Var(ε) = *σ*^2^|*t*|^2*∆*^ where Δ is estimated from the data. Population variation, *b*_*mk*_, was assumed to affect all three parameters. We tested models with population variation in subsets of parameters and with a constant within population variation. The model chosen had the lowest Akaike and Bayesian information criterion [44]. The population variation is assumed independent of the within population variation and also has a normal distribution with zero mean and covariance matrix, **Σ**_*b*_. Parameters of eq. (Z) were estimated by the restricted maximum likelihood techniques implemented by the *nlme* function in R.

#### Development time: larvae to adult

In this experiment, the time from larvae hatching from egg to adult eclosion from pupae was studied. Eggs from the TDO and TSO populations were collected on non-nutritive agar. From each agar plate, 50 first-instar larvae were transferred to polystyrene vials with banana molasses food. Thirteen vials per replicate were assayed. Vials were checked every 6 h after the first adult flies eclosed, and all eclosed flies were counted and sexed by microscope.

Time to eclosion was analyzed using a linear mixed-effects model (LME) in the R-project for statistical computing (www.R.project.org) [43]. The model used for the data is described as follows: Let *y*_*ijkm*_ be the development time for regime – *i* (*i* = 1 (TDO) or 2 (TSO)), sex-*j* (*j* = 1 (female), 2(male), population – *k* (*k* = 1,.., 10) and individual – *m* (*m* = 1,.., n_*jk*_). A LME model for time to eclosion is,

$$ {y}_{ijkm}=\alpha +{\delta}_i{\beta}_i+{\delta}_j\gamma +{\delta}_i{\delta}_j\pi +{b}_k+{\varepsilon}_{ijkm} $$

where *δ*_*s*_ = 0, if *s* = 1, and 1 otherwise, and *b*_*k*_ and ε_*ijkm*_ are assumed to be independent random variables with a normal distribution with zero mean and variances ${\sigma}_1^2$ and ${\sigma}_2^2$ respectively.

#### Adult age-specific fecundity

TDO and TSO adult age-specific fecundity was monitored for 2 weeks. Populations were reared in vials and given 14 days to develop. On day 14 from egg, one mating pair (one male and one female) were transferred to 60 charcoal caps per replicate. Charcoal medium is composed of the following per 1 L distilled H_2_O: 19 g Apex® Drosophila agar type II, 5 g Fisher® Activated Darco® G-60 Carbon, 54 g Sucrose, 32 g Red Star® active dry yeast, 3 g Sigma-Aldrich® Methyl 4-hydroxybenzoate (anti-fungal), and 30 mL EtOH. Starting on day 14, fecundity was monitored every 24 h until day 28. Pairs were given a fresh charcoal cap with 50 μL yeast solution (98 mL distilled water, 2 g active dry yeast, and 2 mL 1% acetic acid) each day, and the old charcoal caps were scanned on a flatbed scanner and counted at a later time.

Age-specific fecundity was analyzed using a linear mixed-effects model (LME) in the R-project for statistical computing (www.R.project.org) [43]. The data consisted of fecundity at an age (*x*) within an age interval − *k* (*k* = 1..,5). Fecundity was modeled by a straight line within each interval. Regime − *j* (*j* = 1 (TDO) or 2 (TSO)) could affect the intercept, but not the slope of the line. Slope could vary between intervals. Populations − *i* (*i* = 1, 2…,10) contributed random variation to these measures. Fecundity at age (*x*), interval (*k*), regime (*j*), and population (*i*) is *y*_*ijkx*_ and can be described by,

$$ {y}_{ijkx}=\alpha +{\beta}_k+{\delta}_j{\gamma}_j+\left(\omega +{\pi}_k{\delta}_k\right)x+{\delta}_k{\delta}_j{\mu}_{jk}+{c}_i+{\mathcal{E}}_{ijkx}, $$

where *δ*_s_ = 0 if *s* = 1 and 1 otherwise, and *c*_*i*_ and ${\mathcal{E}}_{ijkx}$ are independent standard normal random variables with variance ${\sigma}_c^2$ and ${\sigma}_{\mathcal{E}}^2$, respectively. The effects of diet on the intercept are assessed by considering the magnitude and variance of both *γ*_*j*_ and *μ*_*jk*_.

#### Fungal-resistance

Susceptibility to fungal infection was compared between the TDO and TSO populations. The pathogen used was the entomopathogenic fungus *Beauveria bassiana*, strain 12,460 obtained from the USDA Agricultural Research Service Collection of Entomopathogenic Fungi, Ithaca NY. Fungal suspensions were prepared by suspending 0.3 g of *B. bassiana* spores in 25 mL of 0.03% silwet. The TDO and TSO populations were reared in vials and given 12 days to develop. On day 12, the flies were transferred to fresh food vials. On day 14, ~ 500 flies (sexes mixed) were briefly anesthetized with CO_2_ and then placed on Petri Dishes on ice for the duration of the inoculation assay (<2 min). Anesthetized flies were sprayed either with 5 mL of the prepared fungal suspension or with 5 mL of control suspension (0.03% silwet, but not fungus) using a spray tower (Vandenberg 1996). Sprayed flies were then moved to 3 L cages and kept at 100% humidity for 24 h. After 24 h, the humidity was reduced to 60%. Dead flies were removed from the cages daily and were sexed. Food was replaced daily. We completed three technical replicates and tested a total of ~ 1500 flies (sexes mixed) per population per treatment.

Fly mortality, *p*_*ij*_(*t*), was modeled at day-*t* (*t* = 1, 2,..,?) in selection regime-*i* (*i* = 1 (TDO), 2 (TSO)) and treatment-*j* (*j* = 1 (fungus), 2 (no fungus)) by the logistic regression function,

$$ \mathit{\log}\left[\frac{p_{ij}(t)}{1-{p}_{ij}(t)}\right]={\mu}_0+{\delta}_i{\alpha}_0+{\delta}_j{\beta}_0+{\delta}_i{\delta}_j{\gamma}_0+\left({\mu}_1+{\delta}_i{\alpha}_1+{\delta}_j{\beta}_1+{\delta}_i{\delta}_j{\gamma}_1\right)t, $$

where δ_*k k*_ = 1 if *k* = 1 or 0 otherwise. Parameters of this equation were estimated with the *glm* function in R [43].

#### Starvation resistance

On day 15 from egg, 30 female flies from each of the TDO and TSO populations were placed in their own starvation straw, one fly per straw. These starvation straws are capped at both ends and contain a small amount of agar at one end of the straw. This agar “plug” provides adequate humidity, but no nutrients. Mortality was checked every 4 h using lack of movement under provocation as a sign of death.

Female mean longevity in a nutrition free environment was analyzed using a linear mixed-effects model (LME) in the R-project for statistical computing (www.R.project.org) [43]. The model used for the data is described as follows: Let *y*_*ijkm*_ be the longevity for regime – *i* (*i* = 1 (TDO) or 2 (TSO)), population – *j* (*j* = 1,.., 10) and individual – *k* (*k* = 1,.., n_*jk*_). A LME model for longevity is,

$$ {y}_{ijk}=\alpha +{\delta}_i\beta +{b}_j+{\varepsilon}_{ijk} $$

where *δ*_*s*_ = 0, if *s* = 1, and 1 otherwise, and *b*_*j*_ and *ε*_*ijk*_ are assumed to be independent random variables with a normal distribution with zero mean and variances ${\sigma}_1^2$ and ${\sigma}_2^2$ respectively.

#### Desiccation resistance

On day 15 from egg, 30 female flies from each of the TDO and TSO populations were placed in their own desiccant straws, one fly per straw. A piece of cheesecloth separated the fly from the pipet tip at the end of the straw that contained 0.75 g of desiccant (anhydrous calcium sulfate). The pipet tip containing desiccant was sealed with a layer of Parafilm©. Mortality was checked hourly, using lack of movement under provocation as a sign of death.

Female mean longevity in a desiccated environment was analyzed using a linear mixed-effects model (LME) in the R-project for statistical computing (https://www.r-project.org/) [43]. The model is described as follows: Let *y*_*ijkm*_ be the longevity for regime – *i* (*i* = 1 (TDO) or 2 (TSO)), population – *j* (*j* = 1,.., 10) and individual – *k* (*k* = 1,.., n_*jk*_). A LME model for longevity is,

$$ {y}_{ijk}=\alpha +{\delta}_i\beta +{b}_j+{\varepsilon}_{ijk} $$

where *δ*_*s*_ = 0, if *s* = 1, and 1 otherwise, and *b*_*j*_ and *ε*_*ijk*_ are assumed to be independent random variables with a normal distribution with zero mean and variances ${\sigma}_1^2$ and ${\sigma}_2^2$ respectively.

#### Cardiac Arrest Rates

On days 15, 16, and 17 from egg, 30 female flies from each of the TDO and TSO populations were chosen at random (total of 90 flies per replicate). The flies were anesthetized for 3 min using trimethylamine (FlyNap©), and then placed on a microscope slide prepared with foil and two electrodes. FlyNap was chosen as the anesthetic because of its minimal effect on heart function and heart physiology when administered for more than 1 min [45]. The cold-shock method was not used as an anesthetic for the cardiac pacing assay, because the flies need to be fully anesthetized throughout the procedure. If the flies regain consciousness, the added stress and abdominal contractions while trying to escape would alter heart rate and function more than FlyNap does. Paternostro et al. [46] found that FlyNap has the least cardiac disruption compared to the two other substances commonly used for *Drosophila* anesthesia, carbon dioxide and ether.

Two electrodes were attached to a square-wave stimulator in order to produce electric pacing of heart contraction. Anesthetized flies were attached to the slide between the foil gaps using a conductive electrode jelly touching the two ends of the fly body, specifically the head and the posterior abdomen tip. The shocking settings for this assay were 40 V, 6 Hz, and 10 ms pulse duration. Each shock lasted for 30 s. An initial check of the status of the heart was made after completion of the shock, followed by a check after a 2-min “recovery” period. Heart status was scored as either contracting or in cardiac arrest. The protocol for this assay is outlined in Wessells and Bodmer [47].

CMH tests were used to analyze the rates of cardiac arrests between the TSO_1–5_ and TDO_1–5_ populations. The CMH test is used when there are repeated tests of independence, or multiple 2 × 2 tables of independence. Below is the equation for the CMH test statistic, with the continuity correction included, that we used for our statistical analyses:

$$ {X}_{\mathrm{MH}}^2=\frac{{\left\{|\Sigma \left[{a}_i-\frac{\left({a}_i+{b}_i\right)\left({a}_i+{c}_i\right)}{n_i}\right]|-0.5\right\}}^2}{\Sigma \left({a}_i+{b}_i\right)\left({a}_i+{c}_i\right)\left({b}_i+{d}_i\right)\left({c}_i+{d}_i\right)/\left({n}_i^3-{n}_i^2\right)} $$

We designated “a” and “b” as the number of cardiac arrests in the TSO and TDO cohorts of population *i*. We designated “c” and “d” as the number of contracting hearts in these two cohorts of population *i*. The n_*i*_ represents the sum of a_*i*_, b_*i*_, c_*i*_, and d_*i*_. The subscript *i* (*i* = 1..5), representing one of the five replicate populations within the B stock.

### DNA extraction and sequencing

Genomic DNA was extracted from samples of 200 female flies collected from each of the 10 individual populations (TSO_1–5_ and TDO_1–5_) using the Qiagen©/Gentra Puregene© kit, following the manufacturer’s protocol for bulk DNA purification. The 30 gDNA pools were prepared as standard 200–300 bp fragment libraries for Illumina sequencing, and constructed such that each five replicate populations of a treatment (e.g., TSO_1–5_) were given unique barcodes, normalized, and pooled together. Libraries were run across PE100 lanes of an Illumina HiSEQ 2000 at the UCI Genomics Highthroughput Sequencing Facility. Resulting data were 100 bp paired-end reads. Each population was sequenced twice; data from both runs were combined for some analyses as described below. Combining reads from two independent sequencing runs likely alleviate the effects of possible bias introduced from running all replicates for each population in the same lane.

### Genomic analysis

#### Mapping of reads

Reads were mapped to the *D. melanogaster* reference genome (version 6.14) using bwa mem with default settings (BWA version 0.7.8) [48]. The resulting SAM files were filtered for reads mapped in proper pairs with a minimum mapping quality of 20, and converted to the BAM format using the view and sort commands in SAMtools [49]. The rmdup command in SAMtools was then used to remove potential PCR duplicates. As each population was sequenced twice, there were two bam files corresponding to each population at this stage. BAMtools was used to combine pairs corresponding to the same populations [50]. Average coverage was above 70× for all populations except TSO_3_, which was 67× (Additional file 2: Table S1). Next, SAMtools was used to combine the 10 bam files into a single mpileup file. Using the PoPoolation2 software package [51], these files were converted to “synchronized” files, which is a format that allele counts for all bases in the reference genome and for all populations being analyzed. We then used RepeatMasker 4.0.3 (http://www.repeatmasker.org) to create a gff file detailing low complexity regions in the *D. melanogaster* reference genome. The regions were then masked in our sync file once again using PoPoolation2.

#### SNP variation

A SNP table was created using the sync file mentioned above. We only considered sites where coverage was between 30× and 200×, and for a site to be considered polymorphic we required a minimum minor allele frequency of 2% across all 10 populations. All sites failing to meet these criteria were discarded. To assess broad patterns of SNP variation in TSO and TDO populations, heterozygosity was calculated and plotted over 150 kb non-overlapping windows directly from the major and minor counts in our SNP table. A t-test was also performed to compare mean heterozygosity between the two groups of populations. To assess how closely replicate populations resembled one another, F_ST_ estimates were also obtained using the formula: *F*_ST_ = (*H*_T_-*H*_S_)/*H*_T_ where H_T_ is heterozygosity based on total population allele frequencies, and H_S_ is the average subpopulation heterozygosity in each of the replicate populations [52]. *F*_ST_ estimates were made at every polymorphic site in the data set for a given set of replicate populations.

#### SNP differentiation

We used two different methods to assess SNP differentiation in the TSO and TDO populations. First, we used the CMH test as implemented in the PoPoolation2 software package to compare SNP frequencies between the TSO and TDO populations. As the findings of Wiberg et al. [40] indicate that coverage variation can impact statistical results, we subsampled to a uniform coverage of 50× across the genome for each population using scripts provide in the PoPoolation2 software package. During this process, all positions with coverage less than 50× or greater than 200× were discarded. The subsampling procedure involved calculating the exact fraction of the allele frequencies at each site, and linearly scaling them to our target coverage of 50×. In addition to these coverage requirements, we only considered sites polymorphic if they had a minor allele frequency of 2% across all ten populations. In total, the resulting subsampled sync file contained ~ 1.2 million SNPs spread across the major chromosome arms. CMH tests were then performed at every polymorphic site between the TSO and TDO populations. To correct for multiple comparisons, we used the permutation approach featured in Graves et al. [13]. Briefly, populations were randomly assigned to one of two groups and the CMH test was then performed at each polymorphic position in the shuffled data set to generate null distributions of *p*-values. This was done a 1000 times, and each time the smallest *p*-value generated was recorded. The quantile function in R was then used to define thresholds that define the genome-wide false-positive rate, per site, at 5%.

In addition to the CMH test, we also used the quasibinomial GLM approach recommended by Wiberg et al. [40]. Here the authors argue that while the CMH may be the most commonly used test to compare allele frequencies in projects combining experimental evolution and pool-seq, key assumptions of the test are often violated in such studies. For instance, the assumption that each count within a cell of the contingency table being considered by the test is independent is automatically violated in pool-seq studies as counts from reads are not independent draws from the experimental populations being studies (See Wiberg et al. [40] for a more detailed discussion of this issue and others). Based on their findings, the violation of these assumptions results in inflated *p*-values and increased false positive rates. Their findings further suggest the quasibinomial GLM approach they advocate has lower false positive and higher true positive rates than the CMH test. However, it should be noted that the permutation derived significance threshold used in our CMH tests are more stringent than anything featured in their analysis. The test was implemented using scripts provided by Wiberg et al. [40]. A .sync file was once again the primary input file, and we used the same SNP calling criteria outlined above (minimum coverage of 50× per population, maximum of 200× per population, and a minimum minor allele frequency of 2% across all 10 populations). Coverage was once again scaled to 50× to minimize the effect of coverage variation on our results. As counts of zero can lead to problems when implementing this approach, a count of one was added to each allele whenever a zero was encountered. In terms of establishing significance thresholds, another reported benefit of quasibinomal GLMs is that they produce the expected uniform distribution of *p*-values under the null hypothesis which allows for standard method of correcting for multiple comparisons [35]. As a result, to correct for multiple comparisons we used two common approaches, the Bonferroni correction and the *q*-value method [53, 54]. We chose to use the Bonferroni correction and the *q*-value methods as Wiberg et al. [40] found them to be the most and least conservative approach, respectively.

#### Migration simulations

Although all populations are maintained independently, and precautions are taken to prevent accidental migration (eg. vials and cages used for stock maintenance are specifically labeled, a single person is never performing maintenance on the TDO and TSO populations simultaneously, etc.), there have almost certainly been chance migration events over the hundreds of generations these populations have been maintained. As such, it could be argued that the apparent genomic convergence between the TDO and TSO populations following the relaxation of selection for desiccation resistance is due to low frequency migration events. Forward simulations featuring migration were performed to test this idea.

Ideally, simulations would have been done based on SNP frequencies in the TDO and TSO populations at the height of selection for desiccation resistance. However, we do not have such data as these populations were derived well before the rise of next generation sequencing technology, and we have no frozen samples from that time period. As a result, we opted to use data from the ACO and CO populations described in Burke et al. [12] and Graves et al. [13]. The ACO group consists of five replicate populations subjected to selection for accelerated development, while the CO group consists of five replicate populations subjected to selection for delayed reproducing and increased longevity. These different selection regimes have in turn produced significant phenotypic and SNP differentiation between the two groups [12, 13]. Our simulations strategy was to see if 230 generations of drift and low frequency migration were enough to erase the genomic differentiation present between these populations.

In total, 1.1 million SNPs were identified across the five major chromosome arms in the ACO and CO populations (Additional file 9). Based on SNP frequencies at these sites, we generated 5 ACO and 5 CO populations each consisting of 1000 individuals. We chose to simulate 1000 individuals per population as an effective population size of 1000 is supported by past work in our system [41]. To generate haplotypes for individuals in each population, we put 2000 alleles into a pot corresponding to the major and minor allelic frequencies in the real data (eg. Simulated ACO_1_ was generated based on frequencies in the real ACO_1_ population). The pot was shuffled, and alleles were taken out two at a time achieve a random distribution.

To perform our simulations we used MimicrEE2 (https://sourceforge.net/p/mimicree2/wiki/Home/), a forward simulation specifically designed to mimic experimental evolution. It simulates populations of diploid individuals where genomes are provided as haplotypes with two haplotypes constituting a diploid genome. There were no changes in the demography once the initial population file is submitted. The simulated populations have non-overlapping generations and all individuals are hermaphrodites (though selfing is excluded). At each generation, matings are performed, where mating success (number of offspring) scales linearly with fitness, until the total number of offspring in the population equals the targeted population size (fecundity selection). Each parent contributes a single gamete to the offspring. Crossing-over events are introduced according to a user-specified recombination rate. The recombination rates were specified for 100 kb windows and were obtained from the *D. melanogaster* recombination rate calculator v2.232. As recombination does not occurs in male *Drosophila*, the empirically estimated female recombination rate was divided by two for the simulations. From this method, ten populations were generated to match the ten experimental populations.

We used the migration features in MimicrEE2 to see how different levels of migration would impact levels of SNP differentiation in the simulated ACO and CO populations after 230 generations of neutral evolution. We simulated a total of three scenarios: two, six, and ten migration events every generation. For each population, the source of migration was generated using the method outlined above based on the average SNP frequencies across replicates corresponding to a given selection treatment. The ACO populations could only receive CO migrations, while the CO populations could only receive ACO migrants. So, for the two migration event scenario, each generation one of the 5 ACO replicates would receive a CO migrant, and one of the CO replicates would receive an ACO migrant. In the six migration event scenario, each generation three of the 5 ACO replicates would receive a CO migrant, and three of the CO replicates would receive an ACO migrant. Lastly, in the 10 migration event scenario, at each generation every one of the ACO replicates would receive a CO migrant, and every one of the CO replicates would receive an ACO migrant. We then assessed our ability to detect differentiated sites in the resulting simulated data sets using the quasibinomial GLM approach in the same manner it was applied to the TDO and TSO data. We looked at the number of sites detected using the Bonferroni correction (our most stringent method) and the *q*-value approach (our least stringent method).

## Additional files

Additional file 1: Mortality Data. File containing daily mortality data for the TDO and TSO populations. (XLSX 42 kb)

Additional file 2: All supplementary figures and tables for Effects of evolutionary history on genome wide and phenotypic convergence in *Drosophila* populations. (DOCX 4402 kb)

Additional file 3: Time to Eclosion Data. File containing time to eclosion data for the TDO and TSO populations. (XLSX 152 kb)

Additional file 4: Fecundity Data. File containing fecundity data for the TDO and TSO populations. (XLSX 12 kb)

Additional file 5: Fungal Resistance Data. Survivorship and mortality data for TDO and TSO cohorts exposed to the fungal pathogen *Beauveria bassiana*, and control cohorts not exposed to the fungus. (XLSX 19 kb)

Additional file 6: Starvation Resistance Data. Time till death for TDO and TSO individuals subjected to starvation conditions. (XLSX 14 kb)

Additional file 7: Desiccation Resistance Data. Time till death for TDO and TSO individuals subjected to desiccation conditions. (XLSX 14 kb)

Additional file 8: Cardiac Arrest Rate Data. Cardiac arrest rates for TDO and TSO individuals subjected to heart pacing procedure. (XLSX 8 kb)

Additional file 9: ACO and CO SNP Table. A table containing nucleotide counts for all polymorphic sites identified in the ACO and CO populations. This data set was used as the basis for our migration simulations. (ZIP 28661 kb)

## Abbreviations

CMH test: Cochran-Mantel-Haenszel test
LME: Linear mixed-effects model
SNP: Single nucleotide polymorphism
