# Supplementary material for: Effects of evolutionary history on genome wide and phenotypic convergence in Drosophila populations
Source: BMC Genomics. 2018 Oct 11;19:743. doi: 10.1186/s12864-018-5118-7 (PMC6180417; doi:10.1186/s12864-018-5118-7)
Supplement: Supplementary file 2 — All supplementary figures and tables for Effects of evolutionary history on genome wide and phenotypic convergence in Drosophila populations. (DOCX 4402 kb) [file 12864_2018_5118_MOESM2_ESM.docx]

Figures

Figure S1. Survivorship (*l_x_*) for the TDO and TSO populations. TSO populations are represented by red lines and dots. TDO populations are represented by blue lines and dots. Lines represent average survivorship. TDO populations live significantly longer than TSO populations.


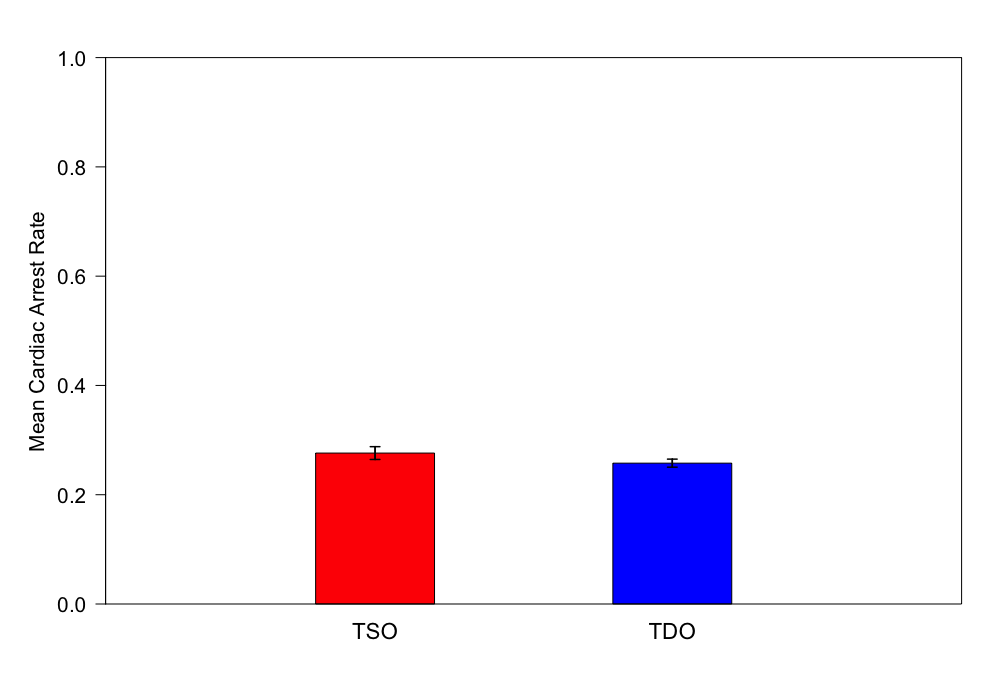


Figure S2. The average rate of cardiac arrests of female fruit flies from the two five-fold replicated stocks (mean ± 1 SEM). There was no statistical difference (p-value = 0.598) in the rates of cardiac arrests between the five TSO (red) and five TDO populations (blue).


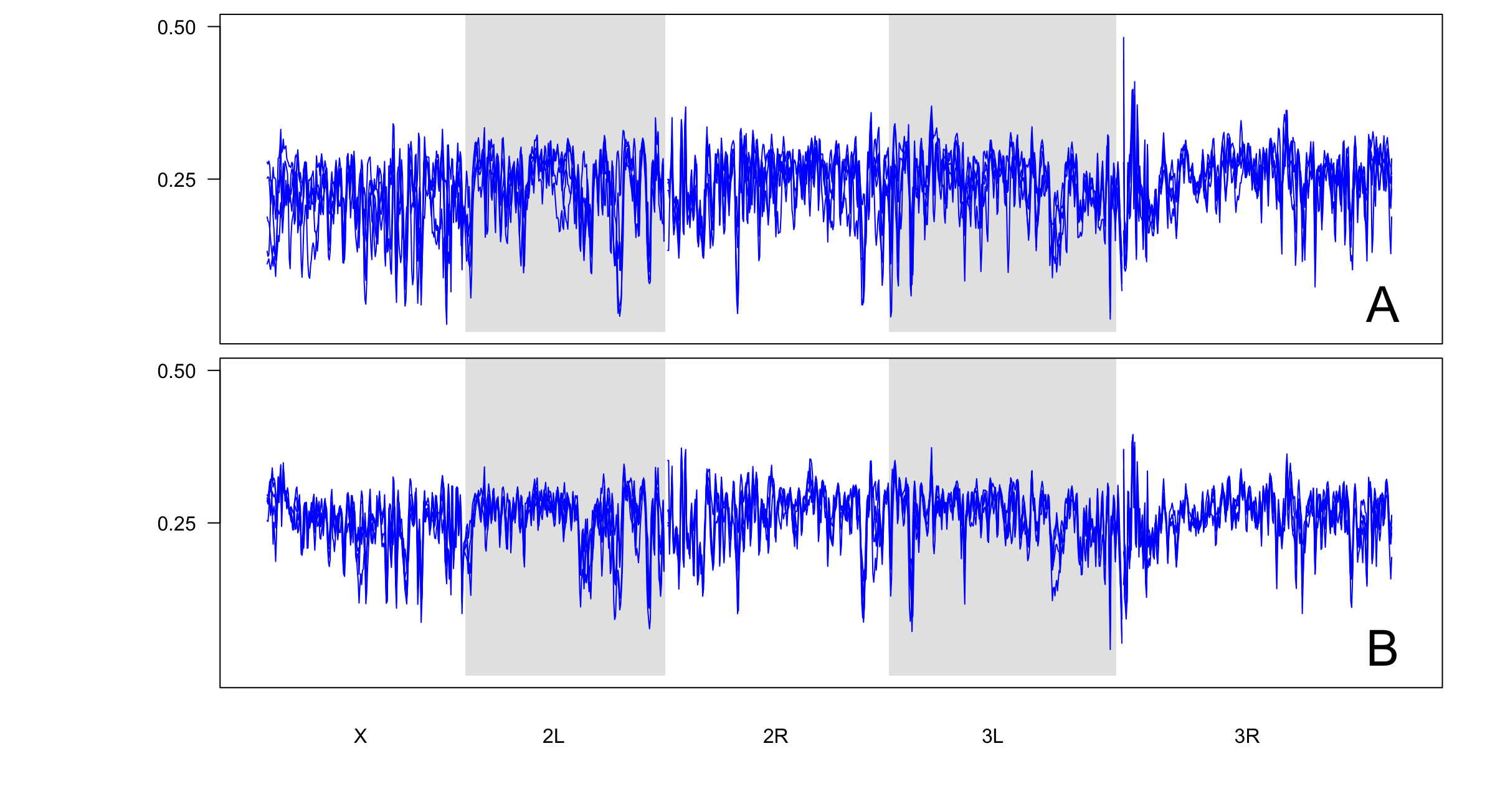


Figure S3. Heterozygosity in the TSO *(A)* and TDO (*B)* populations plotted over 100-kb windows across all major chromosome arms. All replicates are shown for each population.


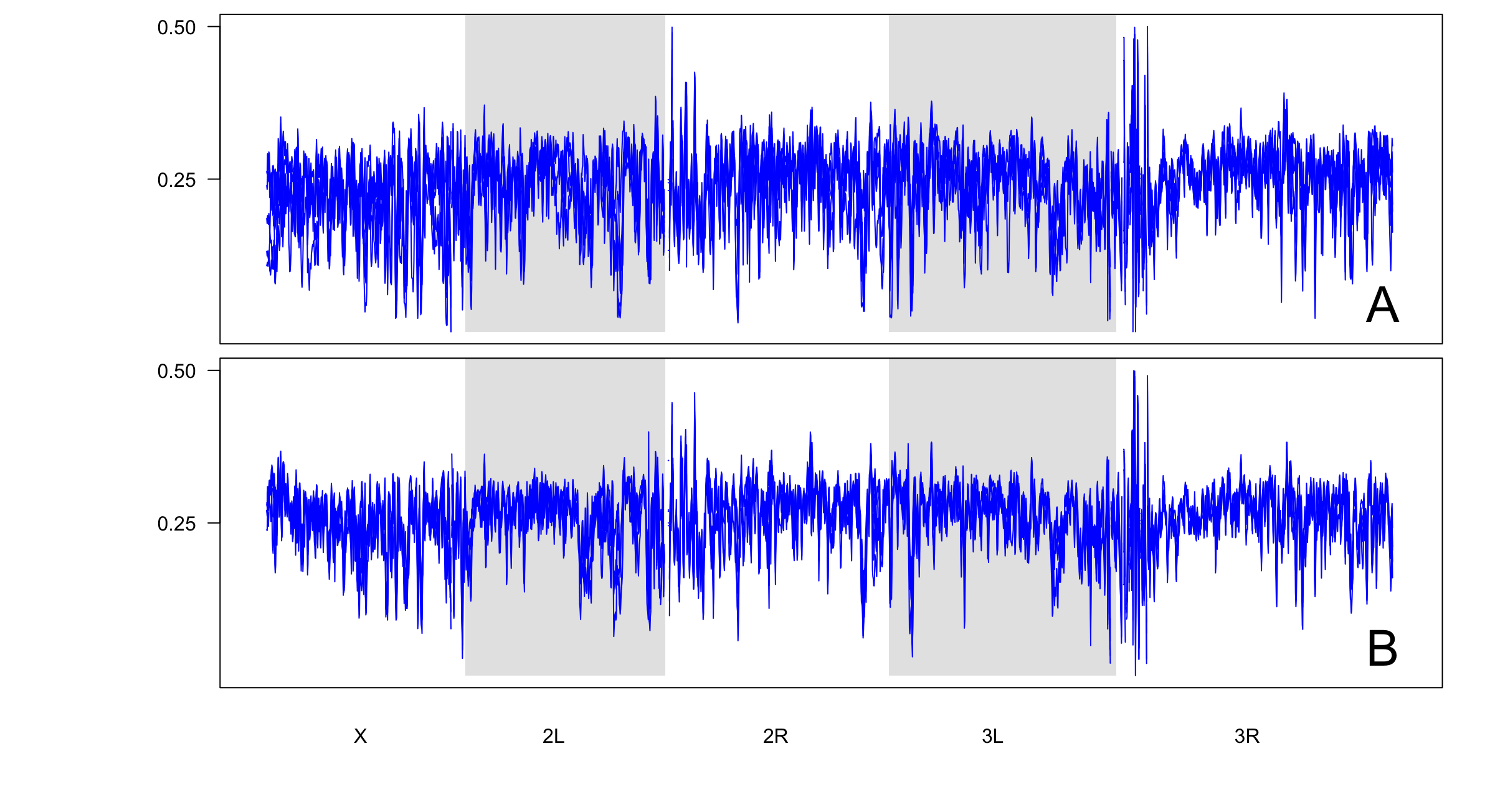


Figure S4. Heterozygosity in the TSO *(A)* and TDO (*B)* populations plotted over 50-kb windows across all major chromosome arms. All replicates are shown for each population.

Figure S5. Results from quasibinomial GLM approach to comparing SNP frequencies plotted as -log(p-values). Results shown for the real ACO vs CO comparison *(A)*, simulated ACO vs. CO data set with 230 generations of drift and 2 migration events per generation *(B)*, simulated ACO vs. CO data set with 230 generations of drift and 6 migration events per generation *(C)*, and simulated ACO vs. CO data set with 230 generations of drift and 10 migration events per generation *(D)*. Bonferroni corrected significance threshold indicated by the red line.


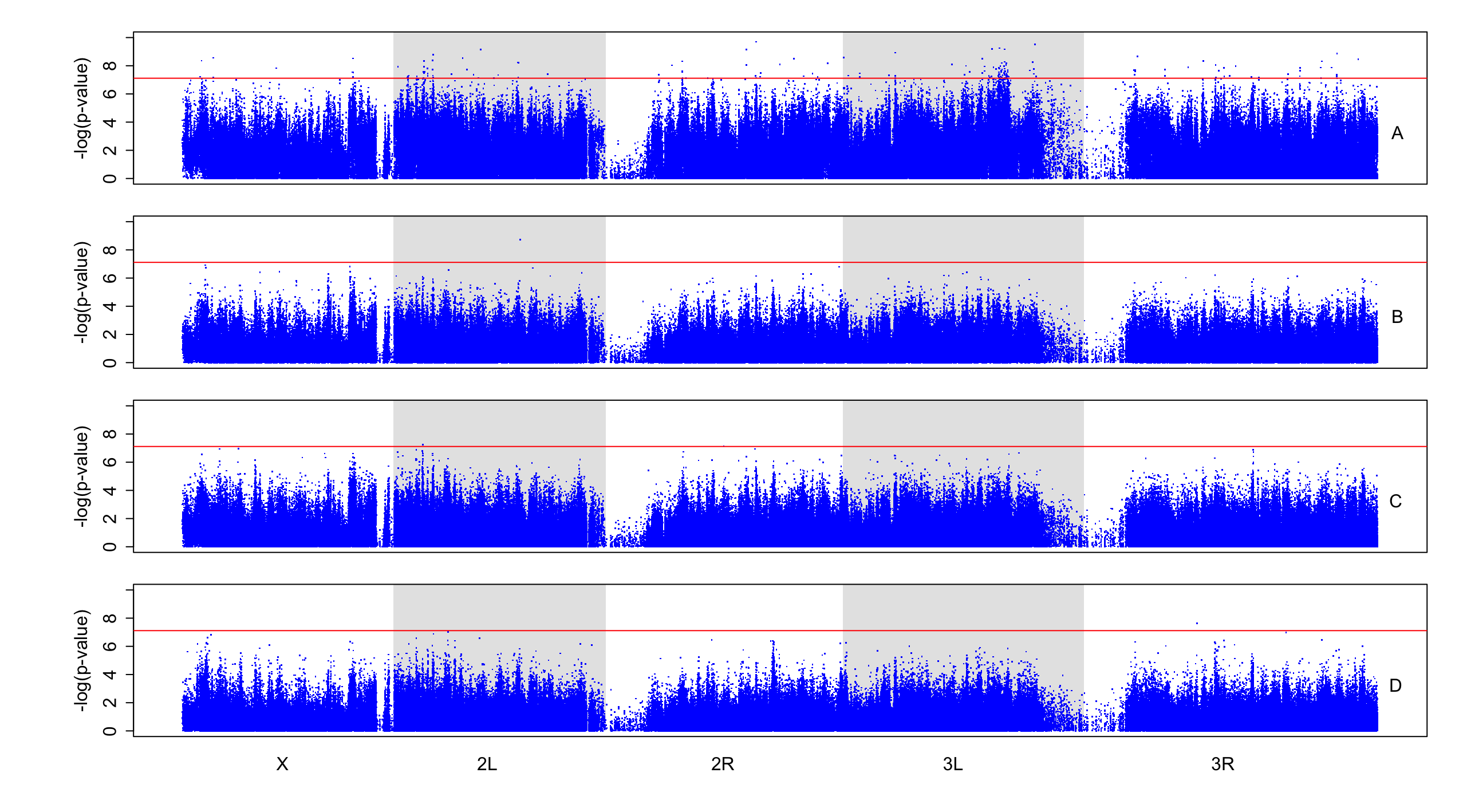


Figure S6. Results from quasibinomial GLM approach to comparing SNP frequencies plotted as -log(q-values). Results shown for the real ACO vs CO comparison *(A)*, simulated ACO vs. CO data set with 230 generations of drift and 2 migration events per generation *(B)*, simulated ACO vs. CO data set with 230 generations of drift and 6 migration events per generation *(C)*, and simulated ACO vs. CO data set with 230 generations of drift and 10 migration events per generation *(D)*. The red line indicates a 0.05 false discovery rate threshold.


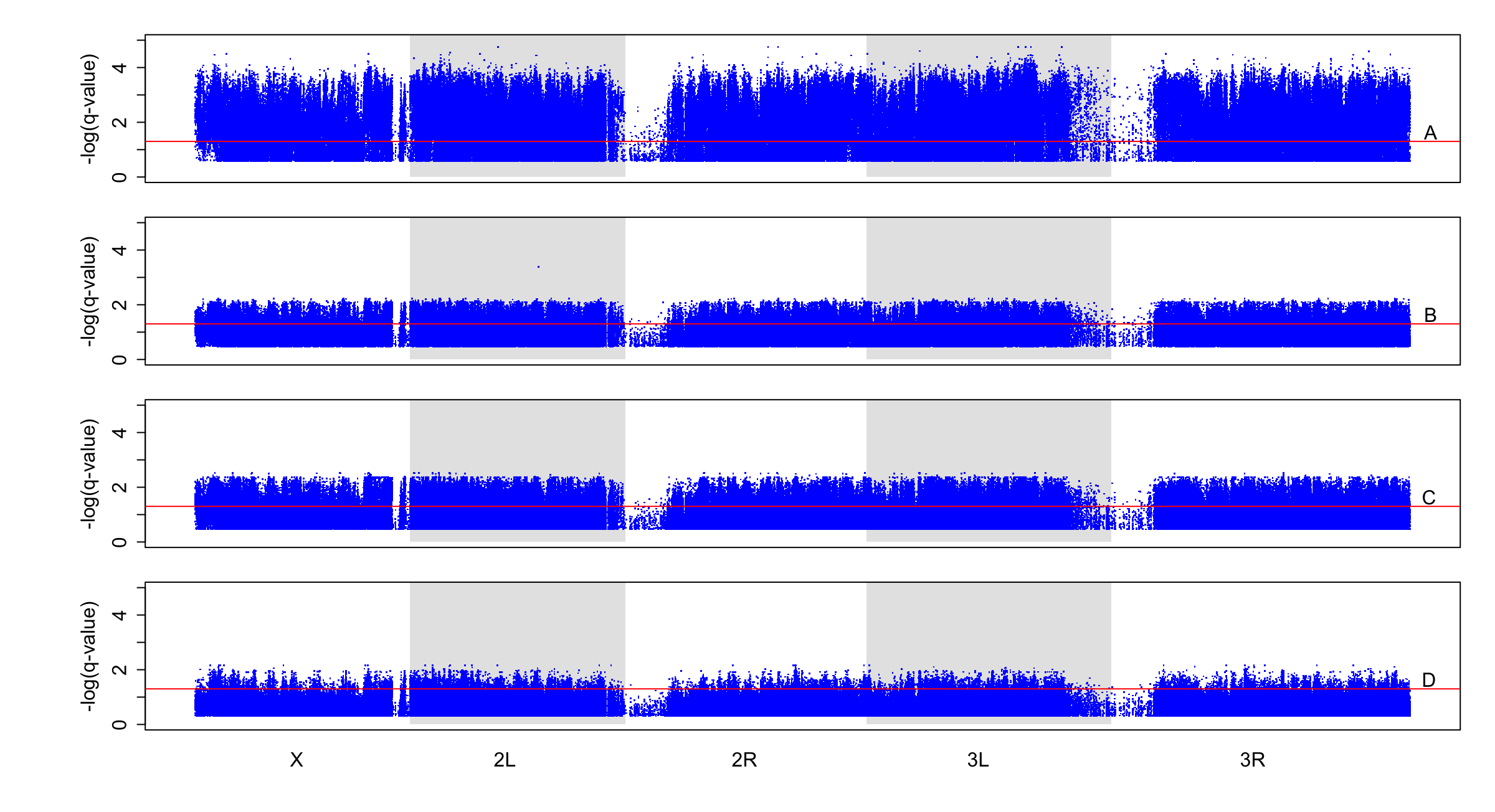


Tables

Table S1. Average read coverage across the genome for all populations used in this study.

| Population | Replicate | Average Read Coverage |
| --- | --- | --- |
| TSO | 1 | 87 |
|  | 2 | 83 |
|  | 3 | 78 |
|  | 4 | 80 |
|  | 5 | 70 |
| TDO | 1 | 96 |
|  | 2 | 81 |
|  | 3 | 67 |
|  | 4 | 87 |
|  | 5 | 78 |

Table S2**.** TSO vs. TDO adult age-specific mortality using the two-stage, three parameter Gompertz model. Significant p-values are bolded.

| Parameter | Difference | Std. error | t-value | D/F | p-value |
| --- | --- | --- | --- | --- | --- |
| *A* | 0.00009 | 0.000022 | 4.0 | 1777 | **0.0001** |
| α | 0.00032 | 0.0047 | 0.07 | 1777 | 0.945 |
| *bd* | -5.73 | 0.927 | -6.18 | 1777 | **<0.0001** |

Table S3**.** TSO vs. TDO mean longevity analysis. Significant p-values are bolded.

| Difference (days) | Std. error | t-value | D/F | p-value |
| --- | --- | --- | --- | --- |
| -6.97 | 1.35 | -5.147 | 8 | **0.0009** |

Table S4**.** TSO vs. TDO mean development time. Significant p-values are bolded.

| Difference (hours) | Std. error | t-value | D/F | p-value |
| --- | --- | --- | --- | --- |
| -1.16 | 2.62 | 0.45 | 8 | 0.66 |

Table S5**.** TSO vs. TDO age-specific fecundity. Significant p-values are bolded.

| Age range | Difference (intercept) | Std. error | t-value | D/F | p-value |
| --- | --- | --- | --- | --- | --- |
| 15-17 | -2.33 | 2.43 | -0.958 | 8 | 0.3663 |
| 18-20 | -6.99 | 2.43 | -2.878 | 8 | **0.0206** |
| 21-23 | -2.29 | 2.43 | -0.944 | 8 | 0.3730 |
| 24-26 | -3.82 | 2.43 | -1.571 | 8 | 0.1549 |
| 27-28 | -1.50 | 2.79 | -0.537 | 8 | 0.6058 |

Table S6**.** TSO vs. TDO mean longevity during starvation. Significant p-values are bolded.

| Difference (hours) | Std. error | t-value | D/F | p-value |
| --- | --- | --- | --- | --- |
| 4.41 | 2.78 | 1.58 | 8 | 0.15 |

Table S7**.** TSO vs. TDO mean longevity during desiccation. Significant p-values are bolded.

| Difference (hours) | Std. error | t-value | D/F | p-value |
| --- | --- | --- | --- | --- |
| -1.77 | 1.16 | -1.16 | 8 | 0.16 |

Table S8. Mean genome-wide heterozygosities calculated from SNP data

|  | TSO | TDO |
| --- | --- | --- |
| Replicate 1 | 0.24 | 0.27 |
| Replicate 2 | 0.25 | 0.27 |
| Replicate 3 | 0.24 | 0.26 |
| Replicate 4 | 0.25 | 0.26 |
| Replicate 5 | 0.26 | 0.27 |
| Mean | 0.25 | 0.27 |

Table S9. Number of significantly differentiated sites detected between the ACO and CO populations, and the number of sites in detected simulated data sets with 230 generations of drift with different migration rates (M).

|  | quasibinomial GLM (Bonferroni correction) | quasibinomial GLM (q-value) |
| --- | --- | --- |
| ACO vs CO (real) | 162 | 425,102 |
| ACO vs CO (M=2) | 1 | 122,789 |
| ACO vs CO (M=6) | 2 | 154,637 |
| ACO vs CO (M=10) | 1 | 20,828 |
